# A Pharmacy Drug Knowledge Assessment Pilot: Who Will Fly Farthest and What Downs the Plane?

**DOI:** 10.3390/pharmacy11030085

**Published:** 2023-05-13

**Authors:** Laura K. Sjoquist, Suzanne M. Surowiec, Jason W. Guy

**Affiliations:** College of Pharmacy, The University of Findlay, Findlay, OH 45840, USA; laura.sjoquist@findlay.edu (L.K.S.); guyj@findlay.edu (J.W.G.)

**Keywords:** pharmacy, education, assessment, drug characteristics, medications

## Abstract

Objective: To evaluate the effectiveness of a sequenced drug knowledge pilot in third professional year students in a capstone course. Methods: A three-phase drug knowledge pilot was conducted in spring 2022. Students completed a total of thirteen assessments, including nine low-stakes quizzes, three formative tests, and a final summative comprehensive exam. Results from the previous year’s cohort (historical control) who only completed a summative comprehensive exam were compared to the pilot (test group) results to assess effectiveness. The faculty spent over 300 h developing content for the test group. Results: The pilot group had a mean score of 80.9% on the final competency exam, which was one percent lower than the control group who had a less rigorous intervention. A sub-analysis was conducted that removed the students who failed (<73%) the final competency exam, and no significant difference in the exam score was found. One practice drug exam was found to be moderately correlated and significant (r = 0.62) with the final knowledge exam performance in the control. The number of attempts on the low-stakes assessments had a low correlation with the final exam score in the test group compared to the control (r = 0.24). Conclusion: The results of this study suggest a need to further investigate the best practices for knowledge-based drug characteristic assessments.

## 1. Introduction

The 2016 Accreditation Council for Pharmacy Education (ACPE) standards establish the need for core curricular components for students to demonstrate as they progress towards degree completion. Key standard updates in 2016 recommend the concept of ‘competency’ as a method for establishing the minimum standards of knowledge to demonstrate adequate ability [1,2,3]. Providing an individualized approach when assessing these minimum knowledge expectations allows for a greater student-centered experience that emphasizes critical thinking and problem solving rather than rote memorization [2]. Standard one of the 2016 standards emphasizes the need for foundational knowledge, including drug characteristics such as drug action and therapeutic use, in addition to implications for biomedical, pharmaceutical, and clinical sciences (for example side effects).

One ACPE-endorsed method for evaluating progression to competence are the entrustable professional activities (EPAs). EPAs are discrete, essential activities and tasks that all new pharmacy graduates must be able to perform without direct supervision upon entering practice. EPAs assist educators in providing clear expectations of student progression to competence in clinical practice and help ensure consistency when identifying whether a student is obtaining the desired minimum level of knowledge and ability [4]. EPAs suggested and endorsed by ACPE within the patient care provider domain emphasizes the importance and application of drug characteristics [3,4].

Mastery learning is an approach to education where learning is treated as an outcome where all students must demonstrate the minimum mastery of a concept [5]. Key principles of mastery learning include: (1) frequent formative assessments used to gauge progress, (2) a minimum passing score, and (3) advancement only once success has occurred [6]. One way to develop the mastery of a concept is through repeated practice and formative feedback such as sequenced low-stakes assessments. Formative assessments are focused on student learning and providing feedback for the growth and development of the learner through informal, low-consequence testing [1,6]. Conversely, summative assessments are more formal, with high-stakes consequences that are designed to determine if the learner is competent on a subject [6]. Formative assessments can take many forms including short quizzes, short writing exercises, case studies, etc. [1]. Some evidence suggests that the utilization of low-stakes quizzing (where students can take quizzes multiple times), improves student performance on summative assessments [7]. However, there is limited evidence on how a series of formative assessments focused on drug characteristics (e.g., brand/generic names, mechanism of action, side effects, etc.) in pharmacy practice may impact student performance on a summative knowledge-based assessment focused on the same topics. The literature is also unclear regarding how many assessments provide the maximum benefit for students without increasing stress and burden.

An additional consideration in determining the optimal assessment frequency is the cognitive load. The internal cognitive load or difficulty of a given task, and external factors such as work, the volume of content, and life responsibilities both affect the ability of the individual to easily obtain knowledge. The learning environment, including the learning materials (for example, the drug characteristic table) and the learning task characteristics (e.g., medication information) affect the extrinsic and intrinsic cognitive load placed on a learner [8]. Providing clear instructions and maintaining consistency in assessments has been shown to reduce the cognitive load, particularly when assessments occur frequently [9,10]. The literature is unclear on the exact number of assessments needed to achieve the mastery of a subject, and is also unclear regarding the threshold of excess. However, some evidence suggests that frequent testing can help improve long-term learning and provides the benefits of formative assessments [11]. However, this must also be balanced with the impacts of increased testing frequency on testing anxiety (i.e., emotional disturbance and worry associated with test taking) as student pharmacists with a high cognitive test anxiety have been shown to test lower and have lower didactic grade point averages as a result [12].

Providing opportunities for students to test their knowledge, reflect on their learning progress, and gain feedback is critical [13]. Additionally, sequenced, recurrent, low-, and moderate-stakes assessments with a consistent structure reduces the cognitive load associated with the inherent large quantity of drug characteristic knowledge necessary for competent pharmacy practice. To identify how best to support student pharmacists and ensure a consistent minimum drug knowledge is being adequately acquired by students prior to their advanced practice experiences, we designed a pilot, drug characteristic assessment series. Therefore, the purpose of this study was to evaluate the effectiveness of a sequential and layered pilot assessing the students’ ability to demonstrate minimum and adequate drug characteristic knowledge.

## 2. Materials and Methods

In line with a key college curricular initiative, educators developed a focused master drug list based upon NAPLEX preparation materials, expert opinion, and drug information databases covering nine therapy domains encompassing four hundred and sixty-one medications. The nine therapeutic domains covered included cardiology, respiratory, endocrine, immunology, infectious disease, gastrointestinal, renal, pain, and neurology and psychiatry. The master drug list contained the brand/generic, therapeutic class, side effects, and warning/contraindications of the top drugs, and was given to third professional year students on the first day of the semester of a didactic capstone course in both spring 2021 (control group) and 2022 (test group). Minor adjustments to improve the clarity, readability, and content were made between the academic years but the lists were kept very similar between the years aside for the increased number of medications given a more scoping review of the NAPLEX preparation materials (364 in 2021 and 461 in 2022, respectively). In addition, review and consensus was completed by three clinical pharmacists on the faculty as well as by clinical instructors responsible for the corresponding content. For example, following review and consensus from the three clinical pharmacist faculty reviewers, the clinical pharmacist on the faculty at our institution responsible for teaching cardiology and who also actively practices in this specialty, reconciled the cardiology medications and information listed in the table provided to students.

A three-part drug knowledge pilot was conducted over the course of the semester. Students completed a total of thirteen assessments, including nine low-stakes quizzes, three moderate-stakes tests, and one final high-stakes comprehensive exam. One 10 question low-stakes multiple choice quiz was assigned per week to the students. The students had an unlimited number of attempts, and the highest score was recorded. Each quiz covered the assigned list of medications specific for a given therapy domain (e.g., all renal meds or all cardiology drugs). One therapy domain was tested each week through these low-stakes quizzes given in our existing learning management system. The same domains were used for the control and test groups. After every three therapy domains were tested, students then took a proctored moderate-stakes formative assessment over the three prior therapy domains consisting of 30 questions each. For example, part 1 tested student pharmacists regarding cardiology, respiratory, and endocrine therapy domains. Questions focused on the brand, generic, therapeutic class, indication, side effects, contraindications, and warnings. Question types included multiple choice, select-all-that-apply, and application-based questions. In total, 840 questions were created for utilization in this drug knowledge pilot. A 60-multiple choice question summative comprehensive drug exam covering all nine domains was then given at the end of the semester to assess student knowledge. To pass the high-stakes summative exam students needed to earn at least a 73%. If students failed the high-stakes summative exam, they were required to remediate the exam, with a maximum of two remediation attempts provided. Once a student successfully remediated, the initial first exam score was counted. If all remediation attempts were unsuccessful, a zero score was given. Results from the control group, who only completed a practice and summative high-stakes exam and did not complete low-stakes quizzes or moderate-stakes assessments, were compared to the pilot groups. The practice and summative high-stakes exam used in the control group also included 60-multiple choice questions covering all nine domains with questions focused on the brand, generic, therapeutic class, indication, side effects, contraindications, and warnings. Exams used in the test group were designed and structured similar to the practice and summative comprehensive drug exam given at the end of the sequence to the control group. All summative exams, including the practice, were given using an external platform with browser lockdown capabilities. Results were analyzed via the t-test in SPSS using a significance level of 0.05. The study was approved by the University institutional review board (#1634).

## 3. Results

**Figure 1:**
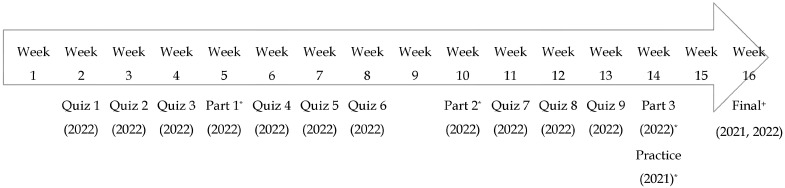
Schematic of the assessment design for the test group (2022) and the control group (2021). * Moderate-stakes proctored assessment, + High-stakes proctored assessment.

Students’ drug knowledge exam scores were compared for the pilot and control groups. Figure 1 depicts the assessment type and frequency between the groups. Table 1 displays the demographics of the two cohort groups. Of the forty-three students who completed the summative high-stakes exam in the control group, six (14%) did not achieve the minimum 73% threshold compared to eight (17%) of the forty-eight students in the test group. The test group completed the low- and moderate-stakes assessments and had a mean score of 80.9% on the final knowledge exam, which was one percent lower than of the control group (*p* = 0.64). A separate analysis was conducted that removed the students who failed (<73%) the final knowledge exam and no significant difference in the exam scores was found (*p* = 0.97). Table 2 displays the comparisons between the control group and the test group. Having one practice drug exam was determined to be moderately correlated with final competency exam performance in the control group, and this correlation was found to be statistically significant (r = 0.62, *p* < 0.001). The number of attempts on the low-stakes assessments had a low correlation with the final high-stakes exam score in the test group (r = 0.24). Additionally, the number of attempts/tries on a low-stakes drug knowledge exam did not have a significant impact on the final exam scores (*p* = 0.24).

Creating and completing the low-, moderate-, and high-stakes knowledge assessments required a significant time investment by both the students and the faculty. Faculty time was tracked, and creating the rigorous competency intervention took approximately 300 h of total time. The perceived student workload was also believed to have increased with the addition of the low- and moderate-stakes assessments in the test group versus only having one high-stakes assessment in the control group (Figure 1).

## 4. Discussion

This study evaluated the effectiveness of a sequenced drug knowledge pilot in third professional year pharmacy students as part of a didactic culminating capstone course. The learning sciences literature indicates that using multiple methods of assessment enhances the validity and fairness of the assessment by providing learners with various ways and opportunities to demonstrate their ability. Furthermore, ideal assessments will measure student progress over time to provide insights into the progression of knowledge acquisition and retention [13]. The pairing of mastery learning with deliberate practice, such as small, low-stakes assessments and practice quizzes, provides learners with systematic and focused feedback as they progress toward competency [14]. Some evidence supports the use of low-stakes assessments and practice quizzes as effective means to assist the students in demonstrating adequate knowledge. The findings of our study, however, are inconsistent with this relationship, and suggest the need for further understanding of the appropriate level and quantity of practice necessary to measure drug characteristic knowledge acquisition. Furthermore, the findings of this study broadly suggest limitations to the effectiveness of this design for measuring student knowledge progression of drug characteristics (e.g., contraindications and side effects). However, the findings from this study are similar to undergraduate education findings with regard to a lack of significant effect on overall student performance compared to the test score performance on low-stakes and moderate-stakes assessments [15]. This study is a first step in investigating how best to assess student competency specifically related to drug characteristic knowledge in a mastery learning design.

Pharmacy and medical literature are unclear regarding the exact number of assessments that are conducive to knowledge acquisition [16]. Given the difference from the baseline testing design in the control group compared to the test group in 2022 (as depicted in Figure 1) cognitive load is certainly necessary to reflect upon. It is possible the high frequency of assessments, although fluctuating in ‘stakes level’, may have contributed to the lack of difference observed in the student performance seen with the pilot structure. While having high rigor testing opportunities aligns with the learning sciences literature and the medical education literature regarding the acquisition of new knowledge, further investigation into the appropriate ‘stakes’ level, volume, and frequency of testing is needed to reconcile the findings of this study with current competency-based evaluation literature [17,18,19,20,21]. Additionally, while the number of drug characteristics required of students was slightly reduced from the control group to the test group, this amount may still have negatively affected the student’s ability to demonstrate their knowledge given these extrinsic factors (for example, employment, other coursework, co-curricular involvements, and leadership roles). Balancing the assessment volume and frequency continues to be a common challenge encountered in pharmacy and medical education, which was equally reflected in the intense pilot design utilized for this study. These items may contribute to test anxiety as learners wrestle to retrieve drug characteristic knowledge within the given timeframe based on the requested information or scenario presented. This additive effect may worsen the negative influence of test anxiety documented in the literature on academic performance, problem-solving abilities, reduced self-efficacy, and decreased perception of self-worth [12,22]. It is also worth noting that the definitive thresholds for competency are unclear in pharmacy and medical education. The 73% benchmark threshold for ‘competency’ set for both the control and test groups may therefore be appropriate or may be too low of a benchmark—as the literature to date is vague. This benchmark was the standard competency threshold utilized by our program.

It may be possible that using quizzes and tests as a method of assessing knowledge in drug characteristics is an inadequate means of evaluating knowledge and may be another explanation for the lack of effects observed in this study. Cumulative assessments are commonplace in US-based pharmacy education, and frequently includes therapeutics, medication counseling, drug information, and pharmacology components [23]. Thresholds for pharmacy curriculum quality recommendations, which encompass drug characteristic knowledge, also vary notably across institutions, something presumed to also be indicative of institutional evaluation thresholds, particularly for cumulative assessments [24]. The lack of an effect seen in the test group in this study calls into question the utilization of a multiple-choice test to assess knowledge. This is supported by criticism of standardized tests by learning scientists [25]. Other assessment models worth evaluating may involve the addition of a practice exam to the tested model, a reduced volume of tests, verbal examination, objective skills testing, and real-world observations to better ascertain knowledge [26,27,28]. The authors believe a multitude of factors influenced the lack of effects seen in this tested design.

There are some implementation challenges to consider. Interestingly, many programs regularly using cumulative evaluation specifically note challenges with determining deficient students, exam validity, buy-in both from student and faculty perspectives, and a lack of evidence regarding exam effects on improved long-term knowledge retention [23]. These concerns are equally notable in this study. Circumstantial factors such as student stress levels, timing of summative assessment (e.g., right before finals week), and personal mental health considerations may also influence student performance [12]. Faculty members and teaching assistants involved in the implementation of this study spent considerable time writing, vetting, overseeing, and assessing the pilot (>350 h, 840 questions). The concept of mental workload from an assessor’s perspective is also less frequently documented in the literature [29,30]. The sustainability and oversight of the assessment structure utilized in this pilot are ongoing concerns expressed by the faculty if the current rigor is maintained, a finding that was found to be consistent with the literature regarding similar cumulative evaluations [23]. Further, the findings of this study suggest opportunities to optimize the use of selective cumulative assessments, and the need to identify ways to improve faculty and student buy-in as well as implementation workload demands.

### 4.1. Limitations

There are a few limitations to this study worth noting. The utility of multiple-choice questions for drug characteristic knowledge assessments are inherently limited. Further, small changes in the content and volume of drug characteristics expected of learners did change minorly from the control group to the test cohort (e.g., duplicate side effects and warnings were listed only once in the test group, whereas duplicates existed in the control group). These changes were minor between the years, and therapy domains were kept consistent. The questions utilized on both the control and test final assessments were kept identical, with only minor grammatical changes made to improve clarity. Different cohorts of students may also limit the comparisons possible between data given inherent differences in class culture, individual study habits, and academic performance. This study also only measured short-term outcomes, and did not assess student performance on clinical rotations, NAPLEX examination, or success in other nonspecific outcomes (e.g., residency placement). Furthermore, grades may not consistently and effectively measure competence or knowledge to the extent desired, however, they have widely been utilized by pharmacy programs as one of the means of providing formative feedback to learners [31]. Finally, this study has limited generalizability given the finite amount of data available and the single institution study design, which thereby may limit the extent to which these findings may apply to other cohorts and institutions.

### 4.2. Future Directions

Additional research is needed to further elucidate the structure and timing of competency-based assessments in order to better support learners in drug characteristic knowledge competency. Blended study designs with quantitative and qualitative measures to assess the effectiveness of smaller, more self-paced drug competencies, as well as adjusted benchmarks to affect student motivation, are needed. The collective experiences of learners and faculty prior to, during competency assessment, and following NAPLEX testing is equally needed to better clarify the needs of pharmacy learners on their journey to drug characteristic knowledge mastery. Based upon the findings of this study, several pilots are currently underway at our institution to re-evaluate alternative competency program structures related to drug characteristic knowledge acquisition, including a revision of this pilot program for future students. Analysis has been planned to determine whether the alternative competency program assessment structures align more closely with current literature findings, and more effectively improve learner outcomes.

## 5. Conclusions

As drug characteristic knowledge continues to remain a critical component of determining learner progression to NAPLEX and advanced pharmacy practice experience readiness, assessment of competency-based drug-characteristic interventions will be critical in illuminating learner abilities [1,3,4]. The assessment design tested in this study (test group) did not prove to be statistically different compared to a more traditional assessment approach (control group). The results of this study suggest that there is a need to further investigate the best practices for competency-based drug-characteristic assessments. Studies comparing multiple assessment methods that utilize qualitative and quantitative metrics are needed to clarify how best to support and document pharmacy learner progression to drug-characteristic readiness.

## Figures and Tables

**Table 1:** Student pharmacist cohort demographics.

Student Cohort	Control—Spring 2021, N (%)	Test—Spring 2022, N (%)
Sample size	43 (100)	48 (100)
Female	30 (69.7)	33 (68.8)
Race		
Caucasian	37 (86)	38 (79)
African American	2 (5)	5 (10)
Other	4 (9)	5 (10)

**Table 2:** Comparison of drug characteristic performance between the groups.

Student Cohort	Control Group Mean (SD)	Test Group Mean (SD)	*p*-Value	95% Confidence Interval
Final summative exam score	81.9 (9.3)	80.9 (10.5)	0.64	1.005 (−3.2, 5.2)
Exam score removing failed first attempts *(i.e., scores <73%)*	84.3 (7.1)	84.3 (7.4)	0.97	0.053 (−3.3, 3.4)

## Data Availability

The data are not publicly available due to University restrictions and student education privacy policies.
